# Terpenoids and Bio-Functions of Essential Oils Hydrodistilled Differently from Freshly Immature and Mature *Blumea balsamifera* Leaves

**DOI:** 10.1155/2023/5152506

**Published:** 2023-03-07

**Authors:** Sirinapha Jirakitticharoen, Wudtichai Wisuitiprot, Pongphen Jitareerat, Chalermchai Wongs-Aree

**Affiliations:** ^1^Division of Postharvest Technology, School of Bioresources and Technology, King Mongkut's University of Technology Thonburi, Bangkok, Thailand; ^2^Department of Thai Traditional Medicine, Sirindhorn College of Public Health Phitsanulok, Phitsanulok, Thailand; ^3^Postharvest Technology Innovation Center, Ministry of Higher Education, Science, Research and Innovation, Bangkok, Thailand

## Abstract

The volatiles and antioxidant capacity of essential oils (EOs) extracted from freshly immature and mature leaves of *Blumea balsamifera* at various hydrodistillation times were investigated. Seven major terpenoids were identified: two monoterpenes, camphor and L-borneol, and five sesquiterpenes, silphiperfol-5-ene, 7-epi-silphiperfol-5-ene, *ß*-caryophyllene, *ɤ*-eudesmol, and *α*-eudesmol. The quantity and terpenoid composition of the EOs were impressed by leaf maturity and hydrodistillation times. The yield of EOs from the immature leaves was 1.4 times that of mature leaves, with 73% of the yield acquired within the first 6 hours (hrs) of hydrodistillation. Approximately 97% of camphor and L-borneol, 80% of *ß*-caryophyllene, silphiperfolene, and 7-epi-silphiperfolene, 32% of *ɤ*-eudesmol, and 54% *α*-eudesmol were collected in the first 6 hrs of hydrodistillation. More *ß*-caryophyllene, *ɤ*-eudesmol, and *α*-eudesmol were found in the mature leaf EOs. The antioxidant capacity of the EOs was proportionally related to their terpenoid contents. The EOs extracted from immature leaves at 0–6 hrs of hydrodistillation demonstrated distinctive antibacterial activity against *Staphylococcus aureus,* with minimum inhibitory concentration (MIC) and minimum bactericidal concentration (MBC) values of 0.5 mg/mL and 1 mg/mL, respectively.

## 1. Introduction

*Blumea balsamifera* (Linn.), a traditional herb locally grown in Thailand, belongs to the family Asteraceae. Based on medical concerns such as cost-effectiveness and widespread use in the local community, its leaves have been utilized in traditional medicine to treat bruising, beriberi, eczema, dermatitis, lumbago, menorrhagia, and rheumatism [1]. In Thailand, the leaves of *B. balsamifera* have long been employed as a flavoring agent in traditional foods and beverages. Furthermore, the leaf extract exhibits free-radical scavenging [2], antiobesity [3], anticancer [4], anti-insomnia activities [5], insecticidal action against the maize weevil (*Sitophilus zeamais*) [6], plasmin inhibition [7], and inhibition of the sympathetic nervous system [5].

*B. balsamifera* leaves have been shown to contain a number of volatile compounds [1], among which are L-borneol, camphor, *ß*-caryophyllene, 10-epi-eudesmol, *ß*-eudesmol, and *α*-eudesmol [8], among other essential oils. Effects of growing conditions and plant maturity on the chemical composition and quantity of EOs from *B. balsamifera* have been explored [9]. *B. balsamifera* growing in the Chinese provinces of Hongshuihe, Luodian, and Guizhou was found to have high quantities of L-borneol. The chemical composition, yield, and antioxidant activity also vary with the plant organs [10]. Camphor, L-borneol, and *ß*-caryophyllene are three primary bioactive chemicals found in *B. balsamifera* leaves, promoting the percutaneous absorption of salbutamol sulfate [11] and having antimutagenic properties [8, 12]. Additionally, research has been conducted to improve extraction efficiency and optimize the preparation and separation of high-purity borneol by modifying the hydrodistillation and sublimation processes [13]. The plant production of *B. balsamifera* has progressively increased in South Asia as a healthy dietary component. Different stages of leaf maturation may accumulate varying levels of bioactive chemicals that may be suited for specific human health purposes [14, 15]. To date, there is no official report, but a bioRxiv preprint [16] on the characteristics of EOs extracted from fresh leaves of *B. balsamifera* at various maturity stages and the extraction efficiency of certain volatiles. Therefore, we explored the volatile compounds in immature and mature leaves of *B. balsamifera* using a hydrodistillation approach at different extraction times and analyzed the EOs for antioxidant and antibacterial activities.

## 2. Materials and Methods

### 2.1. Chemicals

Absolute ethanol was purchased from Daejung (South Korea). Trolox (6-hydroxy-2,5,7,8-tetramethyl-chromane-2-carboxylic acid), DPPH (2,2′-diphenyl-1-hydrazyl), ABTS (2,2′-azinobis-(3-ethylbenzothiazoline-6-sulfonic acid) diammonium salt, and potassium persulfate were supplied by Sigma-Aldrich (USA). *ß*-Caryophyllene was purchased from Tokyo Chemical Industry (Japan). Camphor and endo-borneol were provided by Alfa Aesar (USA). Thiophene was the internal standard for EO analysis (Sigma, USA). All chemicals and reagents were of analytical grade.

### 2.2. Plant Materials

**Figure 1:**
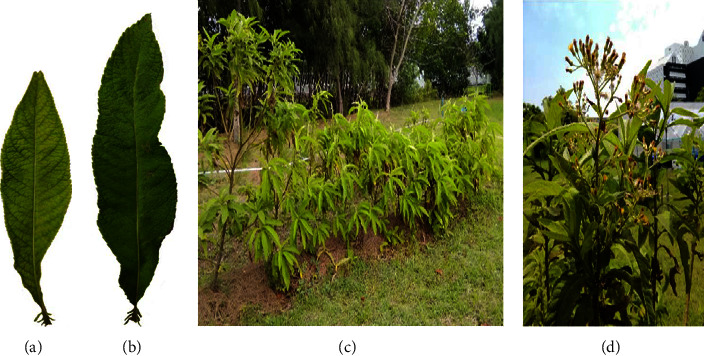
Visual appearances of an immature leaf (a), a mature leaf (b), whole plants of the *B. balsamifera* plant (c), and the plant while flowering (d).

Fresh, bright green immature leaves (the 2^nd^–4^th^ leaves from the shoot, containing small soft trichomes and a soft surface on dorsal *epidermis*) (Figure 1(a)) and dark green mature leaves (containing small stiff trichomes and a matted surface on dorsal *epidermis*) (Figure 1(b)) of *B. balsamifera* were collected in 2019 from 2-year-old plants (Figures 1(c) and 1(d)) cultivated with sufficient water supply in KMUTT Bangkhuntien (N 13.57631; E 100.44295), Bangkok, Thailand. The plant was certified (Voucher Specimen No. ttm-0003856; Crude Drug No. ttm-1000500) by the Thai Traditional Medicine Research Institute, Department of Thai Traditional and Alternative Medicine.

### 2.3. Extraction of Essential Oils

Freshly immature and mature leaves (500 g) were blended and then put in a 10-liter round bottom flask with deionized water (5 L). Next, the flask was subjected to hydrodistillation using a clevenger-type apparatus. Sampling was carried out every 6 hrs until 24 hrs. The recovered EOs were dried over anhydrous sodium sulfate (Figure S1) and stored in sealed vials at −20°C [17] until analysis.

### 2.4. Determination of Volatile Components by GC-MS

Volatile compounds were analyzed using gas chromatography-mass spectroscopy (GC-MS) [18] on an Agilent 6890N (Agilent Technologies, USA) gas chromatograph equipped with an HP-5MS (5% phenyl dimethylpolysiloxane) fused silica capillary column (30 m length × 0.25 mm internal diameter × 0.25 *μ*m film thickness) and an Agilent 5973 Network mass selective detector (Agilent, USA). One *μ*L of the EOs was injected, and helium was used as the carrier gas. The column temperature was initially held at 120°C for 5 mins and then increased to 250°C at 10°C/min. The temperatures of the injector, detector, manifold, and transfer line were 250°C, 200°C, 70°C, and 240°C, respectively, and the ionization energy was 70 eV. The mass spectra ranged from 30–500 amu. The obtained spectra matched to a mass spectral library (the NIST V.14L library values, Palisade Corp., USA) were compared with an internal standard (100 *μ*g/mL thiophene). Camphor, L-borneol, and *ß*-caryophyllene authentic chemicals were subsequently used for quantitative analysis of the compounds. All experiments were analyzed in triplicates.

### 2.5. Determination of Antioxidant Activity

#### 2.5.1. DPPH Radical Scavenging Assay

The free radical scavenging capacity of the EOs was estimated following the DPPH free radical method [19] with slight modifications. The DPPH solution was prepared by dissolving 2.4 mg of DPPH in 100 mL of absolute ethanol. The test solution (50 *μ*L) was added to 1.950 mL of the ethanolic DPPH. The mixture was shaken vigorously and kept at room temperature for 30 min in the dark. The absorbance of the mixture was measured at 517 nm using a UV spectrophotometer (UV-1800, Shimadzu, Japan).

#### 2.5.2. ABTS Radical Scavenging Assay

Free radical scavenging activity of the EOs was also determined using the ABTS radical cation decolorization assay [20]. The ABTS solution was obtained by mixing 7 mM ABTS stock solution with 2.45 mM potassium persulfate at a ratio of 1 : 0.5 and was stored in the dark at room temperature for 12–16 hrs. The ABTS solution was then diluted with absolute ethanol to an absorbance of 0.700 at 734 nm. The *B. balsamifera* extract (50 *μ*L) was mixed with 1.950 mL of the diluted ABTS solution. The absorbance of the mixture was spectrophotometrically measured at 734 nm.

### 2.6. Determination of Antibacterial Activity

The antibacterial activities of EOs extracted from immature leaves at 0–6 hrs and 12–18 hrs of hydrodistillation (Figure S2) were tested *in vitro*. The broth dilution approach described in modified CLSI M7-A7 [21], which is briefly detailed in the next paragraph, was used to determine the minimum inhibitory concentration (MIC) and the minimum bactericidal concentration (MBC). The MIC and MBC of EOs obtained against three different species, namely, *Staphylococcus aureus* (ATCC 6538), *Escherichia coli* (ATCC 8739), and *Pseudomonas aeruginosa* (ATCC 9027), were tested and certified by the Expert Centre of Innovative Herbal Products (Inno Herb), Thailand Institute of Scientific and Technological Research (TISTR).

The inoculums were prepared by growing each of the three bacteria in Mueller-Hinton broth (MHB) at 35 ± 2°C for 18–24 hrs. The bacterial suspensions were adjusted to a turbidity of approximately 1 × 10^8^ CFU/mL, and then 50 *μ*L of each inoculum was put in a tube containing 5 mL of MHB to achieve the final concentrations of each EO in the media. The final concentrations of each EO in MHB were 0, 0.5, 1, and 5 mg/mL. The cultures were incubated at 35 ± 2°C for 18–24 hrs before being assessed for the MIC (Figure S3). Subsequently, the cultures in diluted-EOs broth with zero turbidity (effective inhibition of bacterial growth) were streaked on sterilized MHA plates containing the same concentration of EOs, incubated at 35 ± 2°C for 18–24 hrs, and MBC was determined by observing the presence or absence of bacterial growth (Figure S4). The MBC value was defined as the concentration of EOs in the absence of bacterial colony development.

### 2.7. Statistical Analysis

All experiments were carried out in three replications (3 trees for a replication), and the data were expressed as mean values ± standard deviation. The data were statistically analyzed by variance analysis (ANOVA) at *P* < 0.05–*P* < 0.01 using SPSS software version 18. Mean comparisons were performed using Duncan's multiple range test (DMRT).

## 3. Results and Discussion

### 3.1. Volatile Compounds of Immature and Mature *B. balsamifera* Leaf Extract

**Figure 2:**
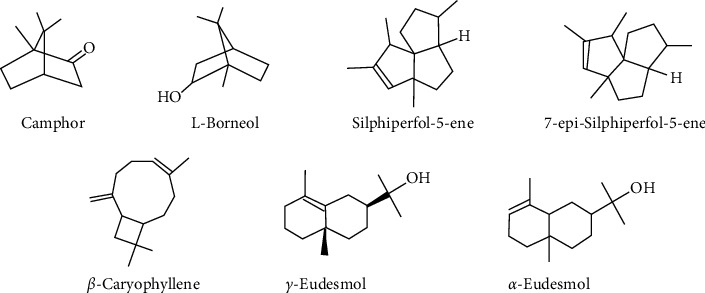
Structure of seven major terpenoids in essential oils from immature and mature leaves of *B. balsamifera*, selected from the GC-MS chromatogram.

The EOs of *B. balsamifera*'s freshly immature, and mature leaves were yellowish in hue (Figures S1 and S5). When compared to mature leaves (352.4 mg/100 g FW), immature leaves produced more EOs (501.9 mg/100 g). The EO yield from immature leaves was 29.8% higher than the amount from mature leaves. According to the research on *Syzygium aromaticum*, a considerable proportion of essential oil is collected in the juvenile stages of leaves, with the largest output being in young leaves (5.1%), followed by expanded leaves (4.5%), expanded leaves (4.1%), and the mature leaves (3.8%) [22]. Previous research has found a link between canopy structure, sunshine exposure, and the resulting plant bioactive compounds [23]. Immature leaves receive more sunlight and acquire more secondary phytochemical compounds due to their outer position, as previously reported in *Zingiber officinale* [24], *Cistus ladanifer* [25], and *Moringa oleifera* [26]. Plants are protected from abiotic and biotic stress by secondary metabolites such as phenolics and flavonoids [27]. Most of the EOs (73%) were extracted during the first 6 hrs of hydrodistillation (Table 1), with the latter stage (18–24 hrs) accounting for only 6.5% of the total yield. The EOs comprised Seven essential volatile compounds, namely, two monoterpenoids, camphor and L-borneol, and five sesquiterpenoids, silphiperfol-5-ene, 7-epi-silphiperfol-5-ene, *ß*-caryophyllene, *ɤ*-eudesmol, and *α*-eudesmol (Table 2, Figure 2, Figures S6–S7). The contents of camphor, borneol, and 7-epi-silphiperfol-5-ene in the EOs from immature and mature leaves were not significantly different (Table 3), even though monoterpenes are biosynthesized in chloroplasts and mature leaves generally contain a higher chlorophyll content. Light has been shown to greatly stimulate monoterpene synthesis in plants while having little effect on sesquiterpene hydrocarbon concentrations [28]. This is probably due to different precursors of exogenous origin in photosynthesis [29]. Moreover, over 90% of the camphor, borneol, sesquiterpenoids of silphiperfolene, 7-epi-silphiperfolene, and *ß*-caryophyllene were eluted from the leaves within the first 6 hrs, whereas only 32.4% of *ɤ*-eudesmol and 54.6% of *α*-eudesmol extracted in the same period.

**Figure 3:**
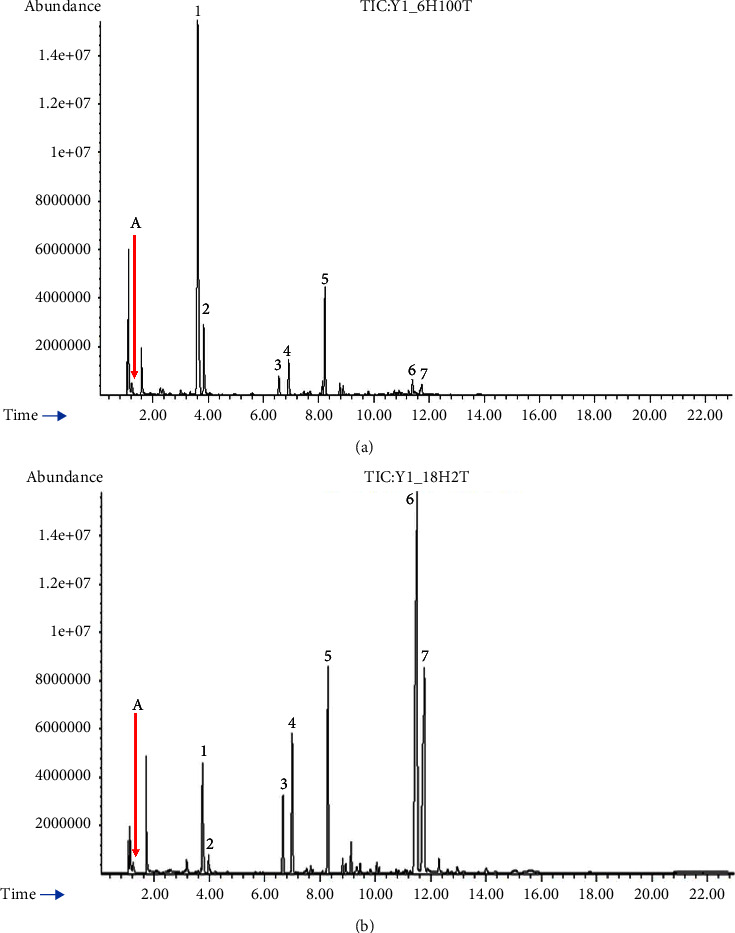
GC-MS chromatographic profiles of seven selected terpenes in essential oils from the immature leaves at 0 − 6 hrs (a) and 12 − 18 hrs (b) of hydrodistillation: (A) internal standard (100 *μ*g/mL thiophene), (1) camphor, (2) L-borneol, (3) silphiperfol-5-ene, (4) 7-epi-silphiperfol-5-ene, (5) caryophyllene, (6) *ɤ*-eudesmol, and (7) *α*-eudesmol.

**Figure 4:**
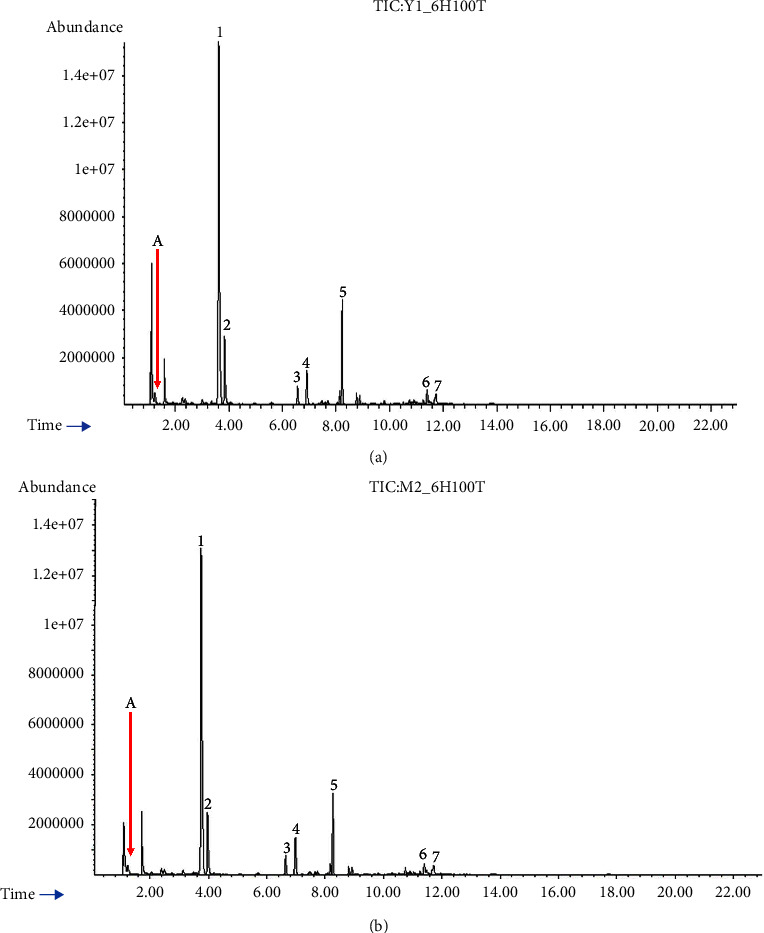
GC-MS chromatographic profiles of seven selected terpenes in essential oils from the immature (a) and mature (b) leaves at 0 − 6 hrs of hydrodistillation: (A) internal standard (100 *μ*g/mL thiophene), (1) camphor, (2) L-borneol, (3) silphiperfol-5-ene, (4) 7-epi-silphiperfol-5-ene, (5) caryophyllene, (6) *ɤ*-eudesmol, and (7) *α*-eudesmol.

Camphor, borneol, and *ß*-caryophyllene contents were subsequently determined using authentic standards that corresponded to the compounds (Table 4). Camphor and borneol are both important ingredients used in traditional Thai medicine [30], while *ß*-caryophyllene is an important sesquiterpene in the pharmaceutical industry for nervous system-related disorders such as depression, pain, anxiety, and Alzheimer's disease [12]. Despite the decline in yield during the later stages of the hydrodistillation, yields of sesquiterpenoids like *ɤ*-eudesmol and *α*-eudesmol remained considerably high at 12–18 hrs (Figure3), in both immature and mature leaves. All eudesmol isomers (*α*-, *ß*-, and *ɤ*-eudesmol) are cytotoxic to cancer cells [31]. Moreover, sesquiterpenoids have a unique tricyclopentane ring structure and a variety of intriguing biomedical and pharmaceutical properties [32]. At 6 hrs of hydrodistillation, the immature leaves' EOs contained more *ß*-caryophyllene and borneol than mature leaves. Camphor was the most prevalent constituent of the EOs, followed by borneol and *ß*-caryophyllene (Figure 4). Other studies have identified L-borneol as the primary component, followed by camphor, *ß*-caryophyllene, eudesmol, isoborneol, and 1,8-cineole [8, 30]. L-borneol, camphor, and *ß*-caryophyllene contents of 42.06, 1.07, and 12.24% were reported for EOs from the leaves of *B. balsamifera* grown in China [10], indicating a possible role of environmental conditions on the chemical composition and yield.

### 3.2. Antioxidant Activity of Extracts from Immature and Mature Leaves

Terpenes and their OH and NH_2_ functional groups of plant volatile compounds have been associated with antioxidant capacity (DPPH and ABTS) [33]. L-borneol, *ɤ*-eudesmol, and *α*-eudesmol containing some OH hydroxyl groups are all greater in immature leaves (5.73, 2.60, and 1.68 g thiophene/100 g FW, respectively) than in mature leaves (4.00, 2.03, and 1.35 g thiophene/100 g FW, respectively). The bioactive compounds have a favorable link with antioxidant capacity, with DPPH capacities of 67.89 g Trolox/100 g FW in immature leaves and 60.39 g Trolox/100 g FW in mature leaves, and ABTS capacities of 224.86 g Trolox/100 g FW in immature leaves and 190.12 g Trolox/100 g FW in mature leaves. The EOs from immature leaves contained higher amounts of these compounds (Table 3) and showed higher DPPH and ABTS activities than EOs from mature leaves, indicating that they were more active (Table 5). Extracts obtained at 0–6 hrs had the highest DPPH and ABTS activities, and the antioxidant activity decreased with extended hydrodistillation periods, mirroring the EOs yield pattern. DPPH is only soluble in organic solvents [34], while ABTS can determine both the hydrophilic and lipophilic antioxidant capacities of samples [35]. Hence, the DPPH value was generally lower, whereas the ABTS assay revealed greater treatment-related differences in antioxidant activity, both in terms of hydrodistillation time and leaf maturity. This implies that the extracted volatile compounds are predominantly lipophilic rather than hydrophilic in nature.

### 3.3. Antibacterial Activity of Extract from Immature Leaves

The antibacterial activity of EOs extracted from immature *B. balsamifera* leaves at 0–6 hrs and 12–18 hrs was determined *in vitro* using three pathogenic bacteria, Gram-positive*S. aureus* (ATCC 6538), Gram-negative *E. coli* (ATCC 8739), and *P. aeruginosa* (ATCC 9027). By 0–6 hrs, the EOs included larger concentrations of camphor, L-borneol, and *ß*-caryophyllene, whereas at 12–18 hrs, *ɤ*-eudesmol and *α*-eudesmol were prevalent (Figure 3). *S. aureus* causes skin and soft tissue infections, bone and joint infections, bacteremia, and endocarditis [36], *E. coli* causes diarrheal disease, sepsis, and urinary tract infections [37], and *P. aeruginosa* causes gastrointestinal infection, keratitis, otitis media, and pneumonia [38]. Both hydrodistillation fractions (0–6 hrs and 12–18 hrs) were effective against *S. aureus*, with MIC values of 0.5 mg/mL and 1 mg/mL, respectively. The broth inoculated with *S. aureus* showed zero turbidity of culture starting from 0.5 to 5 mg/mL of the first 6 hrs fraction, while it was from 1 to 5 mg/mL of 12–18 hrs fraction. All of the concentrations of EOs (0.5–5 mg/mL) were ineffective against *E. coli* and *P. aeruginosa* (Table 6, Figure S3). Sakee et al. [39] reported that leaf-derived EOs of *B. balsamifera* in Thailand showed effective antimicrobial activity against *S. aureus* (1.2 mg/mL MIC)*, Bacillus cereus* (150 *μ*g/mL MIC), and *Candida albicans* (1.2 mg/mL MIC), but there was no effect against *Salmonella enterica, Enterobacter cloacae, Klebsiella pneumoniae, E. coli*, and *P. aeruginosa* [39]. EOs of *B. balsamifera* leaves from Luodian, China, had antibacterial activity against *S. aureus* (9.6 mg/mL MIC) and *E. coli* (4.8 mg/mL MIC), whereas EOs from Hainan, China, inhibited *S. aureus* (2 mg/mL MIC), *C. albicans* (1.2 mg/mL MIC), *P. aeruginosa* (1 mg/mL MIC), and good antifungal activity (62.5–250 *μ*g/mL MIC) [9]. Variation in antimicrobial activity is probably due to differences in the chemical and extraction methods [9]. Furthermore, EOs obtained within the first 6 hrs had an MBC value of 1 mg/mL against *S. aureus* (Table 6, Figure S4). Camphor is a major component of EOs and exhibits the highest aqueous solubility of other terpenoids in EOs [40]. This property probably enables it to penetrate through the outer membrane of bacteria [41]. Gram-negative bacteria are generally more resistant to EOs than Gram-positive ones, which is mainly because the outer layer of Gram-negative bacteria comprises lipopolysaccharides, which restrict the diffusion of hydrophobic compounds through the lipopolysaccharide covering [42].

## 4. Conclusions

From our study, camphor, L-borneol, silphiperfol-5-ene, 7-epi-silphiperfol-5-ene, *ß*-caryophyllene, *ɤ*-eudesmol, and *α*-eudesmol were the primary terpenoids discovered in the essential oils isolated from *B. balsamifera* leaves. In Eos, extracted from immature leaves, *ß*-caryophyllene, *ɤ*-eudesmol, and *α*-eudesmol were found to be more prevalent, but silphiperfol-5-ene was abundant in mature leaves. Camphor, L-borneol, and 7-epi-silphiperfol-5-ene concentrations in essential oils isolated from immature and mature leaves were not significantly different. The majority of the compounds (>90%) were extracted during the first 6 hrs of hydrodistillation, including camphor, borneol, sesquiterpenoids of silphiperfolene, 7-epi-silphiperfolene, and *ß*-caryophyllene, while only 32.4% of *ɤ*-eudesmol and 54.6% of *α*-eudesmol were extracted during this period. At 6 hrs hydrodistillation, the essential oils isolated from immature leaves had the highest antioxidant activity (DPPH and ABTS). The antioxidant capacity was related to the L-borneol, *ɤ*-eudesmol, and *α*-eudesmol contents. EOs extracted from immature leaves after 0–6 hrs of hydrodistillation also showed antibacterial action against *S. aureus*, with MIC and MBC values of 0.5 mg/mL and 1 mg/mL. This study demonstrates that it is advantageous to both alternative and traditional therapies Young leaves of *B. balsamifera* with high bioactive components, antioxidant activity, and antibacterial capabilities are suitable to be chosen for local food or therapy. In addition, 6–hour hydrodistillation of immature leaves can give a significant yield of Eos, and the bioactive chemicals required for further relevant product improvement for modern pharmacists or the cosmetic and food industries.

## Figures and Tables

**Table 1:** Yields of essential oils extracted from immature and mature leaves of *B. balsamifera* with different hydrodistillation periods.

Treatment		Weight (mg/100 g FW)
Maturity	Time	
Immature		501.90 ± 510.48^a^
Mature		352.40 ± 497.65^b^
*F* test (maturity)		^*∗∗*^
	0–6 hrs	1254.45 ± 110.53^a^
	6–12 hrs	222.21 ± 128.01^b^
	12–18 hrs	149.28 ± 88.10^c^
	18–24 hrs	82.64 ± 42.55^d^
*F* test (time)		^*∗∗*^
(Maturity × time)		^*∗*^
Immature	0–6 hrs	1333.67 ± 97.16^a^
	6–12 hrs	336.81 ± 31.61^c^
	12–18 hrs	226.01 ± 10.14^d^
	18–24 hrs	111.10 ± 28.78^e^
Mature	0–6 hrs	1175.24 ± 47.73^b^
	6–12 hrs	107.61 ± 23.89^e^
	12–18 hrs	72.54 ± 40.43^e^
	18–24 hrs	54.19 ± 35.61^e^
*F* test		^*∗∗*^
C.V. (%)		10.83

Data are expressed as mean ± SD (*n* *=* 3). Values in the same column followed by different letters indicate significant differences at *p* < 0.05 according to Duncan's multiple range test; ^*∗*^:  significant at *p* < 0.05 and ^*∗∗*^: significant at *p* < 0.01 level.

**Table 2:** Details of seven major terpenoids in essential oils from immature and mature leaves of *B. balsamifera*, selected from the GC-MS chromatogram of Figures S6-S7.

Compound name^(a)^	RI	MF	RT
(1) Camphor	1121	C_10_H_16_O	3.540
(2) L-borneol	1138	C_10_H_18_O	3.774
(3) Silphiperfol-5-ene	1403	C_15_H_24_	6.512
(4) 7-Epi-silphiperfol-5-ene	1403	C_15_H_24_	6.872
(5) Caryophyllene	1494	C_15_H_24_	8.204
(6) *ɤ*-eudesmol	1626	C_15_H_26_O	11.501
(7) *α*-Eudesmol	1598	C_15_H_26_O	11.747

^a^Compounds listed in order of elution from the HP-5MS column. RI: retention index, n-alkane. MF: molecular formula. RT: retention time.

**Table 3:** Content of seven major terpenoids in essential oils from immature and mature leaves of *B. balsamifera* with different hydrodistillation periods.

Treatment		(g thiophene^1^/100 g FW)						
Maturity	Time	Camphor	L-borneol	Silphiperfol-5-ene	7-Epi-silphiperfol-5-ene	*ß*-caryophyllene	*ɤ*-eudesmol	*α*-Eudesmol
Immature		24.55 ± 43.61^a^	5.73 ± 10.74^a^	1.31 ± 1.88^b^	2.67 ± 1.88^a^	5.70 ± 9.04^a^	2.60 ± 0.80^a^	1.68 ± 1.28^a^
Mature		22.86 ± 39.75^a^	4.00 ± 6.93^a^	1.46 ± 1.94^a^	2.88 ± 1.94^a^	4.56 ± 6.72^b^	2.03 ± 0.54^b^	1.35 ± 0.95^b^
*F* test (maturity)		ns	ns	^*∗*^	ns	^*∗∗*^	^*∗∗*^	^*∗*^
	0–6 hrs	92.31 ± 11.38^a^	18.93 ± 6.82^a^	4.52 ± 0.29^a^	9.11 ± 0.51^a^	18.10 ± 3.13^a^	3.00 ± 0.47^a^	3.31 ± 0.58^a^
	6–12 hrs	2.18 ± 0.57^b^	0.46 ± 0.15^b^	0.64 ± 0.18^b^	1.27 ± 0.33^b^	1.74 ± 0.31^b^	2.59 ± 0.34^b^	0.94 ± 0.09^b^
	12–18 hrs	0.26 ± 0.11^b^	0.06 ± 0.02^b^	0.25 ± 0.05^c^	0.47 ± 0.10^c^	0.48 ± 0.10^c^	2.16 ± 0.74^bc^	1.08 ± 0.37^b^
	18–24 hrs	0.07 ± 0.04^b^	0.01 ± 0.01^b^	0.13 ± 0.04^c^	0.25 ± 0.07^c^	0.20 ± 0.06^c^	1.51 ± 0.29^c^	0.73 ± 0.13^b^
*F* test (time)		^*∗∗*^	^*∗∗*^	^*∗∗*^	^*∗∗*^	^*∗∗*^	^*∗∗*^	^*∗∗*^
(Maturity × time)		ns	ns	ns	ns	^*∗∗*^	ns	ns
Immature	0–6 hrs	95.98 ± 15.96^a^	22.40 ± 8.88^a^	4.41 ± 0.31^a^	9.00 ± 0.64^a^	20.58 ± 2.22^a^	3.39 ± 0.28^a^	3.71 ± 0.56^a^
	6–12 hrs	1.97 ± 0.58^b^	0.47 ± 0.20^c^	0.51 ± 0.03^bc^	1.04 ± 0.07^bc^	1.60 ± 0.17^cd^	2.88 ± 0.17^ab^	0.99 ± 0.04^cd^
	12–18 hrs	0.22 ± 0.14^b^	0.06 ± 0.03^c^	0.23 ± 0.04^d^	0.43 ± 0.08^d^	0.47 ± 0.12^cd^	2.50 ± 0.93^abc^	1.23 ± 0.47^c^
	18–24 hrs	0.04 ± 0.03^b^	0.01 ± 0.00^c^	0.11 ± 0.02^d^	0.20 ± 0.04^d^	0.16 ± 0.05^d^	1.65 ± 0.32^cd^	0.78 ± 0.15^cd^
Mature	0–6 hrs	88.64 ± 5.34^a^	15.46 ± 1.19^b^	4.64 ± 0.25^a^	9.21 ± 0.44^a^	15.62 ± 1.02^b^	2.61 ± 0.17^abc^	2.90 ± 0.15^b^
	6–12 hrs	2.40 ± 0.59^b^	0.45 ± 0.11^c^	0.77 ± 0.17^b^	1.50 ± 0.34^b^	1.87 ± 0.39^c^	2.29 ± 0.11^cd^	0.90 ± 0.11^cd^
	12–18 hrs	0.30 ± 0.08^b^	0.06 ± 0.01^c^	0.27 ± 0.06^cd^	0.52 ± 0.10^cd^	0.50 ± 0.11^cd^	1.83 ± 0.43^bcd^	0.94 ± 0.23^cd^
	18–24 hrs	0.09 ± 0.04^b^	0.02 ± 0.01^c^	0.16 ± 0.03^d^	0.29 ± 0.06^d^	0.23 ± 0.05^cd^	1.36 ± 0.23^d^	0.67 ± 0.14^d^
*F*-test		^*∗∗*^	^*∗∗*^	^*∗∗*^	^*∗∗*^	^*∗∗*^	^*∗∗*^	^*∗∗*^
C.V. (%)		25.13	65.12	11.36	11.04	17.11	24.99	18.93

Data are expressed as mean ± SD (*n* *=* 3) (^1^calculated by comparing to thiophene). Values in the same column followed by different letters indicate significant differences at *p* < 0.05 according to Duncan's multiple range test; ns: not significant, ^*∗*^: significant at *p* < 0.05, and ^*∗∗*^: significant at *p* < 0.01 level.

**Table 4:** Quantitative analysis of camphor, L-borneol, and *ß*-caryophyllene in essential oils from immature and mature leaves of *B. balsamifera* with different hydrodistillation periods.

Treatment		(*μ*g^1^/100 g FW)		
Maturity	Time	Camphor	L-borneol	*ß*-caryophyllene
Immature		147.89 ± 261.24^a^	58.72 ± 104.95^a^	22.18 ± 35.35^a^
Mature		140.12 ± 242.80^a^	45.34 ± 77.08^a^	19.02 ± 29.11^b^
*F* test (maturity)		ns	ns	^*∗∗*^
	0–6 hrs	559.13 ± 63.44^a^	199.40 ± 52.28^a^	73.80 ± 8.60^a^
	6–12 hrs	14.43 ± 3.19^b^	7.12 ± 1.16^b^	6.31 ± 0.76^b^
	12–18 hrs	1.88 ± 0.63^b^	1.15 ± 0.14^b^	1.61 ± 0.27^c^
	18–24 hrs	0.57 ± 0.24^b^	0.44 ± 0.04^b^	0.68 ± 0.15^c^
*F* test (time)		^*∗∗*^	^*∗∗*^	^*∗∗*^
(Maturity × time)		ns	ns	^*∗∗*^
Immature	0–6 hrs	576.11 ± 91.83^a^	225.87 ± 68.22^a^	80.48 ± 6.87^a^
	6–12 hrs	13.35 ± 3.30^b^	7.44 ± 1.60^c^	6.06 ± 0.42^cd^
	12–18 hrs	1.66 ± 0.79^b^	1.16 ± 0.22^c^	1.58 ± 0.31^de^
	18–24 hrs	0.42 ± 0.16^b^	0.43 ± 0.02^c^	0.59 ± 0.12^e^
Mature	0–6 hrs	542.16 ± 27.64^a^	172.93 ± 8.87^b^	67.11 ± 1.92^b^
	6–12 hrs	15.50 ± 3.33^b^	6.81 ± 0.74^c^	6.55 ± 1.04^c^
	12–18 hrs	2.11 ± 0.46^b^	1.13 ± 0.04^c^	1.64 ± 0.29^de^
	18–24 hrs	0.72 ± 0.22^b^	0.46 ± 0.05^c^	0.76 ± 0.14^e^
*F*-test		^*∗∗*^	^*∗∗*^	^*∗∗*^
C.V. (%)		23.57	46.76	12.42

Data are expressed as mean ± SD (*n* *=* 3) (^1^calculated by comparing to the authentic standard). Values in the same column followed by different letters indicate significant differences at *p* < 0.05 according to Duncan's multiple range test; ns: not significant and ^*∗∗*^: significant at *p* < 0.01 level.

**Table 5:** Antioxidant capacities of essential oils from immature and mature leaves of *B. balsamifera* with different hydrodistillation periods.

Treatment		(*μ*g trolox/100 g FW)	
Maturity	Time	DPPH	ABTS
Immature		67.89 ± 6.41^a^	224.86 ± 41.64^a^
Mature		60.39 ± 7.29^b^	190.12 ± 46.17^b^
*F*-test (maturity)		^*∗∗*^	^*∗∗*^
	0–6 hrs	70.96 ± 4.78^a^	276.24 ± 18.31^a^
	6–12 hrs	63.80 ± 5.99^b^	200.22 ± 21.81^b^
	12–18 hrs	62.92 ± 8.41^b^	180.92 ± 26.74^c^
	18–24 hrs	58.87 ± 7.36^b^	172.57 ± 19.51^c^
*F*-test (time)		^*∗*^	^*∗∗*^
(Maturity × time)		ns	Ns
Immature	0–6 hrs	72.48 ± 4.90^a^	288.23 ± 17.37^a^
	6–12 hrs	67.55 ± 4.51^ab^	219.76 ± 4.16^c^
	12–18 hrs	67.12 ± 10.81^ab^	203.66 ± 15.11^cd^
	18–24 hrs	64.40 ± 3.68^ab^	187.78 ± 13.79^de^
Mature	0–6 hrs	69.44 ± 5.12^ab^	264.25 ± 10.26^b^
	6–12 hrs	60.05 ± 5.22^bc^	180.68 ± 5.13^e^
	12–18 hrs	58.71 ± 2.66^bc^	158.18 ± 2.88^f^
	18–24 hrs	53.35 ± 5.52^c^	157.37 ± 8.26^f^
*F*-test		^*∗*^	^*∗∗*^
C.V. (%)		8.98	5.24

Data are expressed as mean ± SD (*n* *=* 3). Values in the same column followed by different letters indicate significant differences at *p* < 0.05 according to Duncan's multiple range test; ns: not significant, ^*∗*^:significant at *p* < 0.05, and ^*∗∗*^: significant at *p* < 0.01 level.

**Table 6:** The minimum inhibitory concentration (MIC) and minimum bactericidal concentration (MBC) of essential oils from immature leaves of *B. balsamifera* extracted by hydrodistillation at the period of 0–6 hrs and 12–18 hrs against *in vitro* growth of *S. aureus, E. coli,* and *P. aeruginosa*.

Period time	No. of repetitions	MICs of the extracts against the tested strains (mg/mL)			MBCs of the extracts against the tested strains (mg/mL)		
		*S. aureus*	*E.coli*	*P. aeruginosa*	*S. aureus*	*E.coli*	*P. aeruginosa*
0–6 hrs	1	0.5	>5	>5	1	>5	>5
	2	0.5	>5	>5	1	>5	>5
	3	0.5	>5	>5	1	>5	>5

12–18 hrs	1	1	>5	>5	>5	>5	>5
	2	1	>5	>5	>5	>5	>5
	3	1	>5	>5	>5	>5	>5

## Data Availability

The authors confirm that the data supporting the findings of this study are available within the article and its supplementary material. Raw data that support the findings of this study are available from the corresponding authors upon reasonable request.

## Supplementary Materials

Supplementary Materials Figure S1: visual appearance of immature leaf hydrodistilled extracts obtained at 0–6 hrs (A), 6–12 hrs (B), 12–18 hrs (C), and 18–24 hrs (D). Figure S2: 100 mg/mL immature leaf EOs obtained at 0–6 hrs (left) and 65 mg/mL EOs obtained at 12–18 hrs (right). Figure S3: The inoculum cultures of *Staphylococcus aureus* (left), *Escherichia coli* (middle), and *Pseudomonas aeruginosa* (right) in Mueller–Hinton broth (MHB) containing 0–6 hrs (A) and 12–18 hrs (B) immature leaf extracted EOs after incubation for 18–24 hrs at 35 ± 2°C. The concentrations of EOS in each tube are as follows: (1) 0 mg/mL, (2) 60% ethanol, (3) 0.5 mg/mL, (4) 1.0 mg/mL, and (5) 5.0 mg/mL. Figure S4: evaluation for an MBC by observing the absence of bacterial growth of *Staphylococcus aureus* on solid agar o MHA containing 0–6 hrs (A) and 12–18 hrs (B) immature leaf EOs. Figure S5: hydrodistillation apparatus and EOs extracted from *B. balsamifera*'s fresh leaves. Figure S6: GC-MS chromatogram profile of seven chosen terpenes ((1) camphor, (2) L-borneol, (3) silphiperfol-5-ene, (4) 7-epi-silphiperfol-5-ene, (5) caryophyllene, (6) *ɤ*-eudesmol, and (7) *α*-eudesmol) in essential oils extracted from immature leaves during a period of the first 6 hrs (100-fold diluted). Figure S7: GC-MS chromatogram profile of seven chosen terpenes oils ((1) camphor, (2) L-borneol, (3) silphiperfol-5-ene, (4) 7-epi-silphiperfol-5-ene, (5) caryophyllene, (6) *ɤ*-eudesmol, and (7) *α*-eudesmol) in essential extracted from immature leaves during a period of 6 to 12 hrs (10-fold diluted).
