# Supplementary material for: Terpenoids and Bio-Functions of Essential Oils Hydrodistilled Differently from Freshly Immature and Mature Blumea balsamifera Leaves
Source: J Trop Med. 2023 Mar 7;2023:5152506. doi: 10.1155/2023/5152506 (PMC10014153; doi:10.1155/2023/5152506)
Supplement: Supplementary Materials — Figure S1: visual appearance of immature leaf hydrodistilled extracts obtained at 0–6 hrs (A), 6–12 hrs (B), 12–18 hrs (C), and 18–24 hrs (D). Figure S2: 100 mg/mL immature leaf EOs obtained at 0–6 hrs (left) and 65 mg/mL EOs obtained at 12–18 hrs (right). Figure S3: The inoculum cultures of Staphylococcus aureus (left), Escherichia coli (middle), and Pseudomonas aeruginosa (right) in Mueller–Hinton broth (MHB) containing 0–6 hrs (A) and 12–18 hrs (B) immature leaf extracted EOs after incubation for 18–24 hrs at 35 ± 2°C. The concentrations of EOS in each tube are as follows: (1) 0 mg/mL, (2) 60% ethanol, (3) 0.5 mg/mL, (4) 1.0 mg/mL, and (5) 5.0 mg/mL. Figure S4: evaluation for an MBC by observing the absence of bacterial growth of Staphylococcus aureus on solid agar o MHA containing 0–6 hrs (A) and 12–18 hrs (B) immature leaf EOs. Figure S5: hydrodistillation apparatus and EOs extracted from B. balsamifera's fresh leaves. Figure S6: GC-MS chromatogram profile of seven chosen terpenes ((1) camphor, (2) L-borneol, (3) silphiperfol-5-ene, (4) 7-epi-silphiperfol-5-ene, (5) caryophyllene, (6) ɤ-eudesmol, and (7) α-eudesmol) in essential oils extracted from immature leaves during a period of the first 6 hrs (100-fold diluted). Figure S7: GC-MS chromatogram profile of seven chosen terpenes oils ((1) camphor, (2) L-borneol, (3) silphiperfol-5-ene, (4) 7-epi-silphiperfol-5-ene, (5) caryophyllene, (6) ɤ-eudesmol, and (7) α-eudesmol) in essential extracted from immature leaves during a period of 6 to 12 hrs (10-fold diluted). [file 5152506.f1.docx]

***Supporting Information***


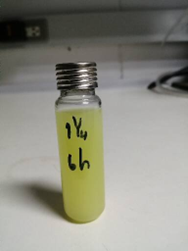

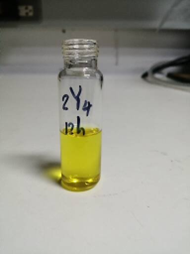

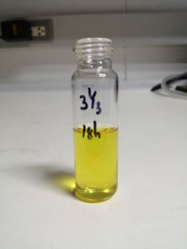

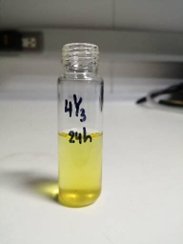


**A B C D**

Figure S1**:** Visual appearance of immature leaf hydrodistilled extracts obtained at 0−6 hrs (A), 6−12 hrs (B), 12−18 hrs (C), and 18−24 hrs (D)


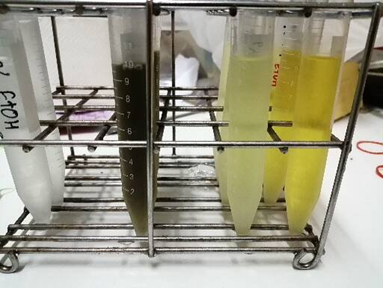


Figure S2: 100 mg/mL immature leaf EOs obtained at 0−6 hrs (Left) and 65 mg/mL EOs obtained at 12−18 hrs (Right).

**
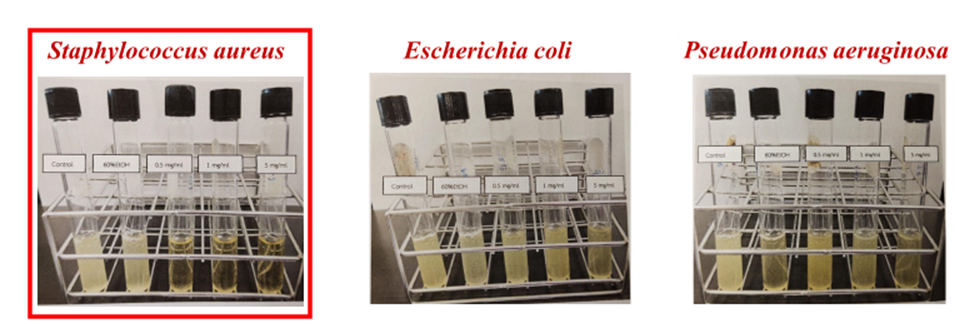
**

**A**

**
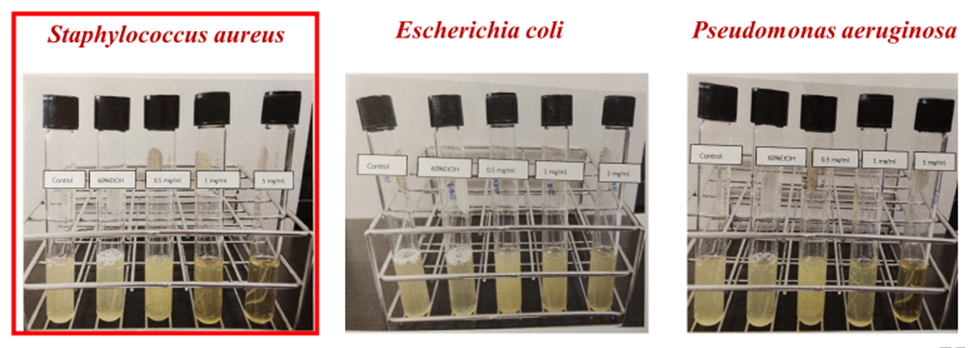
**

**B**

Figure S3: The inoculum cultures of *Staphylococcus aureus* (Left), *Escherichia coli*  (Middle), and *Pseudomonas aeruginosa* (Right) in Mueller-Hinton broth (MHB) containing 0−6 hrs (A), and 12−18 hrs (B) immature leaf extracted EOs after incubation for 18−24 hrs at 35±2 ºC. The concentrations of EOS in each tube: 1) 0 mg/mL, 2) 60% ethanol, 3) 0.5 mg/mL, 4) 1.0 mg/mL, and 5) 5.0 mg/mL

**
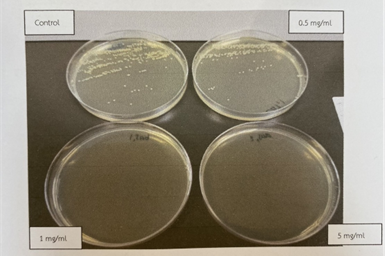
**

**A**

**
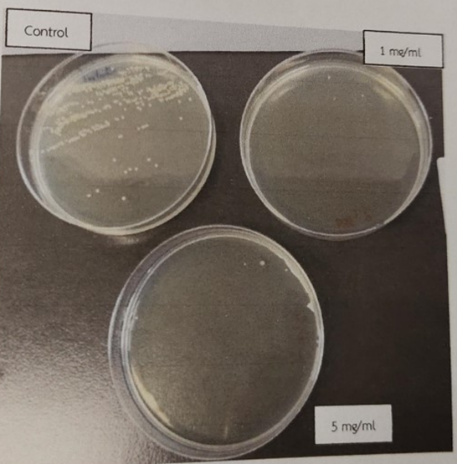
**

**B**

Figure S4: Evaluation for an MBC by observing the absence of bacterial growth of *Staphylococcus aureu* on solid agar o MHA containing 0−6 hrs (A) and 12−18 hrs (B) immature leaf EOs


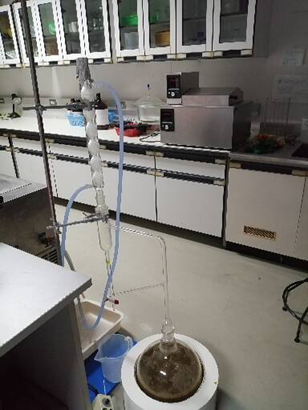


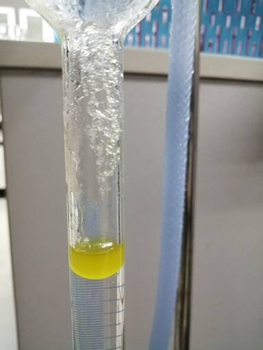

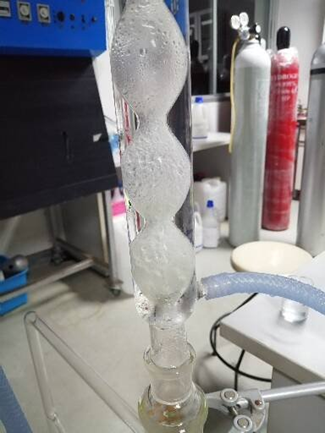

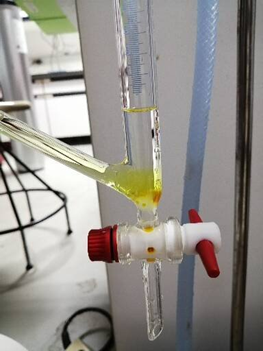


Figure S5: Hydrodistillation apparatus and EOs extracted from *B. balsamifera*'s fresh leaves

.


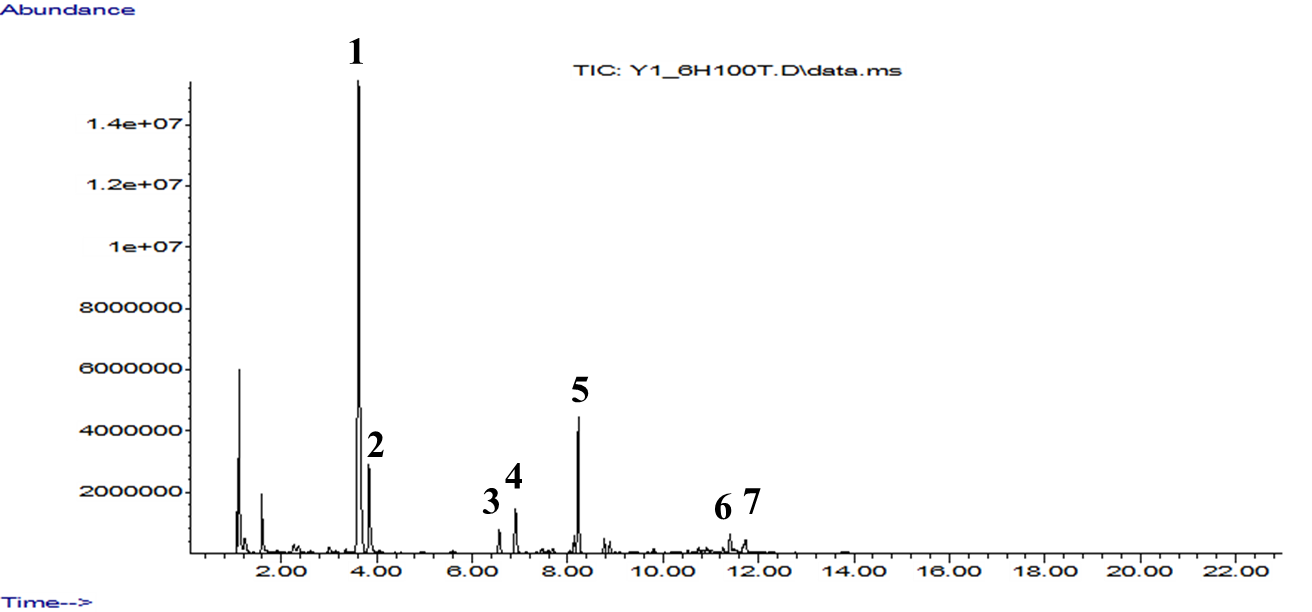


Figure S6: GC-MS chromatogram profile of seven chosen terpenes [ 1) camphor, 2) L-borneol, 3) silphiperfol-5-ene, 4) 7-epi-silphiperfol-5-ene, 5) caryophyllene, 6) ɤ-eudesmol, 7) α-eudesmol] in essential oils extracted from immature leaves during a period of the first 6 hrs (100-fold diluted).


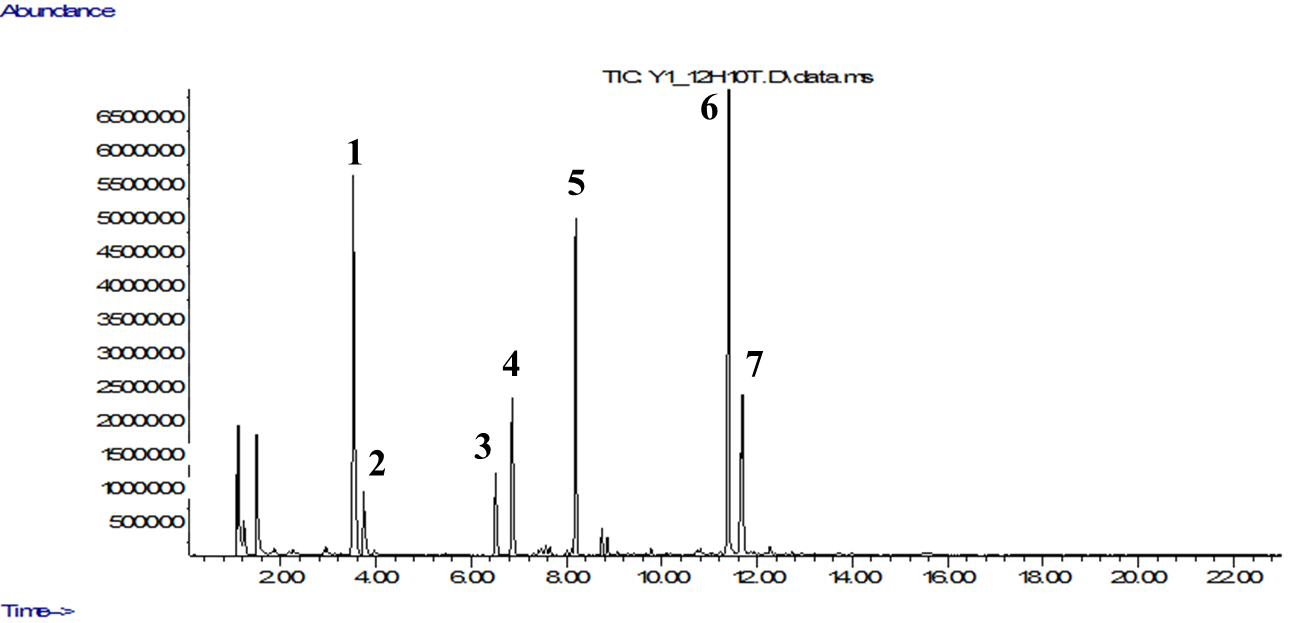


Figure S7: GC-MS chromatogram profile of seven chosen terpenes oils [ 1) camphor, 2) L-borneol, 3) silphiperfol-5-ene, 4) 7-epi-silphiperfol-5-ene, 5) caryophyllene, 6) ɤ-eudesmol, 7) α-eudesmol] in essential extracted from immature leaves during a period of 6 to 12 hrs (10-fold diluted).
